# Dietary quercetin and vitamin E supplementation modulates the reproductive performance and antioxidant capacity of aged male breeder chickens

**DOI:** 10.1016/j.psj.2022.101851

**Published:** 2022-03-11

**Authors:** Felix Kwame Amevor, Zhifu Cui, Zifan Ning, Gang Shu, Xiaxia Du, Ningning Jin, Xun Deng, Dan Xu, Yaofu Tian, Yao Zhang, Diyan Li, Yan Wang, Xiaohui Du, Xiaoling Zhao

**Affiliations:** ⁎ Farm Animal Genetic Resources Exploration and Innovation Key Laboratory of Sichuan Province, Sichuan Agricultural University, Chengdu, Sichuan Province 611130, China; † Department of Basic Veterinary Medicine, Sichuan Agricultural University, Chengdu, , Sichuan Province 611130, China

**Keywords:** aged male chicken, antioxidant capacity, quercetin, spermatogenesis, vitamin E

## Abstract

Aged male chickens experience rapid declines in spermatogenesis, antioxidant capacity, immunity, and hormone synthesis. Vitamin E plays a significant role in reproduction, nervous system function, and disease resistance in animals. Quercetin also exerts many biological effects, such as antioxidant ability, immunostimulation, and protection of spermatozoal plasma membranes. This study evaluated the effects of combining dietary quercetin (**Q**) and vitamin E (**VE**) on sperm quality, antioxidant capacity, immunity, and expression of genes related to spermatogenesis, immunity, apoptosis, and inflammation in aged male chickens. A total of 120 Tianfu breeder male chickens (65 wk old) were randomly allotted to 4 treatments with 3 replicates (10 birds each). The birds were fed diets containing Q (0.4g/kg), VE (0.2g/kg), **Q+VE** (0.4g/kg + 0.2g/kg), and a basal diet for 11 wk. At the end of the experimental period, blood, semen, liver, testes, and spleen samples were collected from 2 birds per replicate. Serum hormones, antioxidant parameters, cytokines, and immunoglobulins were evaluated; and the mRNA expression of genes related to spermatogenesis, apoptosis, and inflammation are determined in the testes and liver tissues.

The results showed that the combination quercetin and vitamin E significantly promoted the sperm count and motility, as well as elevated the levels of testosterone, follicle-stimulating hormone, and luteinizing hormone, antioxidant enzymes (Superoxide dismutase, Glutathione, and Total antioxidant capacity), and serum immunoglobulins (**IgA** and **IgM**) in the aged male chickens; also Q+VE showed protective effects on the liver against injury. In addition, **Q+VE** significantly increased the expression of genes related to spermatogenesis (*AR, pgk2, Cyclin A1, and Cyclin A2*), immunity (***IFN-γ*** and ***IL-2***), and anti-inflammatory cytokines (***IL-10***) (*P* < 0.05), whereas the expression of proinflammatory cytokines (***IL-1β*** and ***IL-6***) was decreased (*P* < 0.05). Taken together, these data indicate that the combination of quercetin and vitamin E improved reproductive characteristics such as spermatogenesis, sperm quality, and hormone regulation, as well as promoted antioxidant defense, hepatoprotective capacity, and immune response in aged male chickens without any detrimental effects.

## INTRODUCTION

Fertility is important for both mammals and birds. However, many factors such as the environment, diet, age, minerals, and vitamins can affect fertility (Nawab et al., 2019). Fertility in male breeder chickens, declines at approximately 45 wk of age (Safari et al., 2018). A reduction in the reproductive performance of broiler breeders is significantly associated with chronological age. This multifactorial problem is influenced by several factors such as a decline in hormone secretion, the development of testicular tissue, diet, and physical body composition (Sarabia et al., 2013). The challenges of meeting up with the high demand for chicken products by humans could be resolved by improving the fertility of chickens even during aging periods to ensure high productivity throughout the year. This can be achieved by promoting reproductive hormone secretion, high-quality semen production, high libido, and mating capabilities, consequently improving the reproductive performance of aging male breeder chickens (Anggorodi, 1994).

Several functional genes are involved in testicular development, spermatogenesis, and sperm characteristics in animals (Payne and Hales, 2004). For instance, ***CYP17*** is highly expressed in the Leydig cells of the testicles of numerous vertebrate species (Payne and Hales, 2004). ***CYP17A1*** is found in the smooth endoplasmic reticulum of Leydig cells and is implicated in the catalysis of 2 important functional oxidase reactions: the transformation of progesterone to 17*α*-hydroxyprogesterone by 17*α*-hydroxylase and the subsequent change from 17*α*-hydroxyprogesterone to androstenedione by 17, 20-lyase. **17HSDs** promotes steroidogenesis (Ye et al., 2011). They are involved in catalysis of the last step in the biosynthesis of active gonadal steroid hormones (estradiol and testosterone) (Payne and Hales, 2004). Moreover, ***17HSD2*** was reported to be encoded by ***HSD17B2*** and is capable of catalyzing testosterone and androstenedione interconversion, whereas ***17HSD3*** is highly expressed in testicular tissue (Wu et al., 1993).

Nutrition is an essential factor that promotes the reproductive efficiency of animals. Therefore, dietary manipulation is an effective method to improve the reproductive potential of male animals (Anggorodi, 1994). Quercetin (3,3,4,5,7- pentahydroxyflavone) is a major dietary polyphenolic flavonoid found in a variety of beverages, fruits, and vegetables with diverse biological activities including phyto-estrogenic, immunoregulatory, anti-inflammatory, and antiviral activities; it also inhibits lipid peroxidation, thereby protecting against oxidative injury and cell death (Yang et al., 2018). Quercetin can promote the function of estradiol (**E_2_**) and other related hormones, hence it is widely used as a supplement in poultry diets to improve egg production and immunity in laying hens (Liu et al., 2013; Yang et al., 2018; Amevor et al., 2021). Quercetin attenuates the reproductive toxicity of 4-nitrophenol to restore the intracellular antioxidant system in testicular cells of embryonic chickens (Mi et al., 2010). Another study by Zhang, 2005 reported that quercetin protected cultured chicken spermatogonial cells from aroclor 1254-induced oxidative damage (Mi and Zhang, 2004).

Vitamin E (**VE**) is a hydrophobic, peroxyl radical-trapping, chain-breaking antioxidant found in the lipid fraction of living organisms. Its core role is to protect the lipid materials of an organism by preserving the integrity of tissue structures from the unwelcome effects of uncontrolled, spontaneous autoxidation (Abedi et al., 2017; Liu et al., 2019). In the poultry industry, vitamin E is usually supplemented as α-tocopherol, which is the most abundant and bioavailable form (Dalia et al., 2018). It has been reported that antioxidants such as vitamin E are useful in restoring the balance between reactive oxygen species (**ROS**) generation and scavenging activities, and can also boost male reproductive performance (Adewoyin et al., 2017). Vitamin E supplementation facilitates liver function and acts as an anti-stressor in chickens (Bollengier et al., 1998; Puthpongsiriporn et al., 2001; Ciftci et al., 2005). Dietary vitamin E supplementation has been reported to improve semen characteristics in male Japanese quail (Biawas et al., 2007). Hooda et al. (2007) reported that adult male quails receiving moderate supplemental vitamin E (75 and 150 IU/kg) had a higher cloacal gland index, testicular weight and plasma testosterone than quails fed a VE-deficient diet (Hooda et al., 2007).

Although, few studies have reported the individual effects of quercetin or vitamin E on the reproductive performance of male animals, to the best of our knowledge, the synergistic effects of combining quercetin and vitamin E is yet to be explored, especially in aged male chickens. Based on previous studies, we hypothesized that a dietary combination of quercetin and vitamin E would improve semen quality, antioxidant capacity, immunoglobulins, and anti-inflammatory cytokines, as well as act as hepatoprotective agents in aging male chickens. Therefore, the objective of this study was to investigate the effects of a dietary combination of quercetin and vitamin E on the production performance, immunity, redox balance, and reproductive attributes of aged male breeder chickens.

## MATERIALS AND METHODS

### Ethics Statement

This study was approved by the Animal Care and Use Committee of Sichuan Agricultural University. Animals used in this experiment were cared for under the guidelines stated in the Guide for the Care and Use of Agricultural Animals in Agricultural Research and Teaching of Sichuan Province, China (No. 2019502005).

### Experimental Animals, Design, and Management

A total of 120 male Tianfu Commercial Broilers (65 wk old) obtained from the Chicken Breeding Unit, Sichuan Agricultural University were randomly divided into 4 treatments, 3 replicates of 10 male chickens each. Quercetin (95%, High-Performance Liquid Chromatography [**HPLC**]) and vitamin E were supplied by Shanxi Huike Plant Development Co., Ltd. (Xian, China). The purity of quercetin was determined by HPLC. The chickens were fed basal diets (Control group); basal diet supplemented with 0.4 g/kg Quercetin powder (Quercetin group); basal diet supplemented with 0.2 g/kg Vitamin E (Vitamin E group); and basal diet supplemented with the combination of 0.4 g/kg Quercetin and 0.2 g/kg Vitamin E (Q + VE group). The recommended levels of vitamin E and quercetin were chosen based on previous studies Yang et al. (2020, 2018) and Liu et al. (2013), respectively.

**Figure 1:**
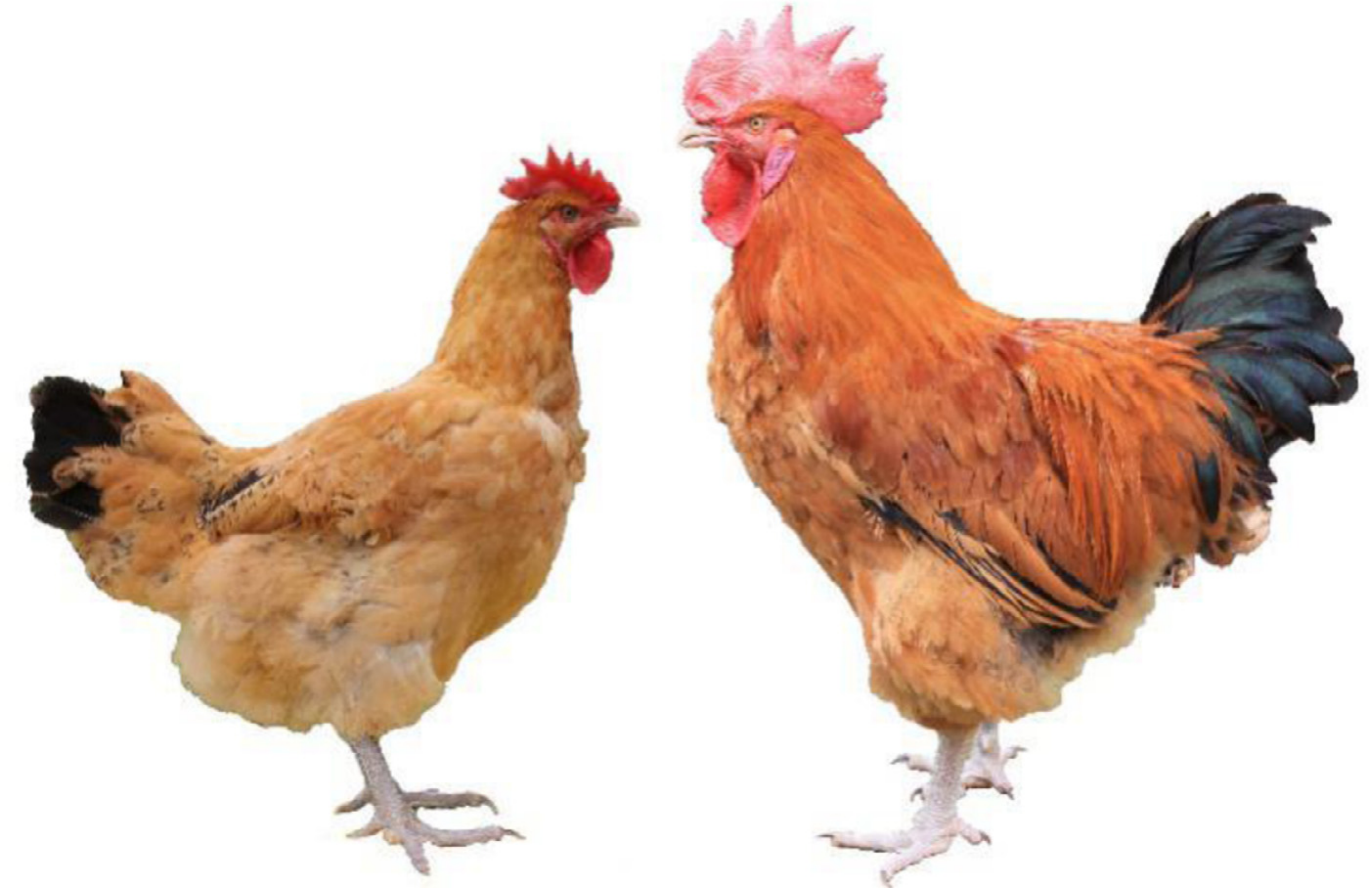
Tianfu Commercial Broiler Breeder Chickens.

Tianfu Commercial Broiler Breeder Chicken is a high-quality meat-type chicken produced by the Poultry Research Breeding Group of Sichuan Agricultural University and Sichuan Banghe Agricultural Science and Technology Co., Ltd. of China. Their market age is 70 d, and the average body weight of the male and female are 2.5 and 2.0 kg, respectively (Amevor et al., 2021; Tian et al., 2021). Their images are shown in Figure 1.

The composition and nutritional values of the basal diet are presented in Table 1 (Amevor et al., 2021). Throughout the 11-wk experimental period, weekly and daily measurements were taken on the chicken's body weight and feed intake, respectively.

**Table 1:** The composition and nutritional values of the basal diet (% dry matter).

Ingredient	Content (%)	Nutrient	%
Corn	56.4	Metabolic energy (ME)	10.98 MJ/Kg
Soybean meal	26.2	Crude protein	17.04
Wheat bran	2.4	Crude fat	3.47
Corn germ meal	3.5	Crude fibre	2.68
Lard	0.8	Calcium	3.4
Limestone (fine)	2.3	Total phosphorus	0.63
Limestone (coarse)	6	Available phosphorus	0.37
Dicalcium phosphate	1.34	Lysine	0.86
Sodium chloride	0.24	Methionine	0.39
Choline chloride	0.12		
Vitamin premix^1^ + mineral premix^2^	0.7		
Total	100		

1 Vitamin premix supplied (per kg of diet): Vitamin A, 6,000 IU; Vitamin D_3_, 1,500 IU; Vitamin K_3_, 4.2 mg; Vitamin B_1_, 3 mg; Vitamin B_2_, 10.2 mg; Vitamin E, 0.0 mg, Folic acid, 0.9 mg; Calcium pantothenate, 15 mg; Niacin 45 mg; Vitamin B_6_, 5.4 mg; Vitamin B_12_, 24 μg; Biotin 150 μg.

2 Mineral premix provided (per kg of diet): Cu (CuSO_4_·5H_2_O), 6.8 mg; Fe (FeSO_4_·7H2O), 66 mg; Zn (ZnSO_4_·7H_2_O), 83 mg; Mn (MnSO_4_·H_2_O), 80 mg; I (KI), 1 mg; Se (Na_2_SeO_3_), 0.3 mg.

### Sample Collection and Procedure

Semen and blood samples were collected at the end of the 11th wk. Blood samples (5 mL) were obtained from 2 male chickens per replicate via the wing vein. Thereafter, they were centrifuged at 3,000 rpm for 10 min at 4°C to collect the serum, and then stored at −80°C for further analyses. Subsequently, 2 chickens per replicate were euthanized and their liver, spleen, and testicular samples were collected, weighed, and snap-frozen in liquid nitrogen and stored at −80°C for subsequent RNA extraction and qRT-PCR analysis. Liver tissues were stored in 4% paraformaldehyde solution for subsequent histological and morphological analyses.

### Evaluation of Semen Characteristics

Immediately after semen collection, the semen volume was measured in a graduated collection tube. Thereafter, the semen was diluted (1:100 in 0.9% NaCl) and used to evaluate sperm motility, viability, and concentration using a computer-aided semen analysis system (ML-608JZII; Nanning Songjingtianlun Bio-technology Co., Ltd, Guangxi, China). Sperm abnormality (calculated as the percentage of abnormal spermatozoa in the total spermatozoa analyzed) was determined by in vivo staining with crystal violet (Santiago-Moreno et al., 2009). After staining, the slides were air-dried and examined under a light microscope (Olympus, Tokyo, Japan) at 400 × magnification. The semen volume, sperm motility, and total sperm in the ejaculate (sperm count) were determined as follows: semen volume (mL) was measured as the volume of semen collected in one ejaculation; sperm count was measured as the number of sperm per milliliter (mL) of semen in one ejaculation and sperm motility (%) was determined as the percentage of sperm that had normal movement.

### Immune Organs Index, Immunological Assays, Antioxidant Parameters, and Hormones Determination

The immune organ index was calculated as the spleen organ weight divided by the body weight. Serum immunoglobulin M (**IgM**) and IgA levels were determined using commercially specific enzyme-linked immunosorbent assay (**ELISA**) kits following the manufacturer's instructions (Nanjing Jiancheng Bioengineering Institute, Nanjing, China). The levels of testosterone, follicle-stimulating hormone (**FSH**), Luteinizing hormone (**LH**), aspartate aminotransferase (**AST**), and alanine aminotransferase (**ALT**) in the serum were determined using kits from Nanjing Jiancheng Bioengineering Institute. In addition, antioxidant parameters (T-SOD, MDA, GSH, and T-AOC) were determined in the serum, liver, and semen using commercially specific biochemistry kits following the manufacturer's instructions of Nanjing Jiancheng Bioengineering Institute. Furthermore, the levels of total cholesterol (**TC**) and triglycerides (**TG**) were determined in the serum and liver using commercial biochemistry kits according to the manufacturer's instructions (Nanjing Jiancheng Bioengineering Institute). Protein concentration in the tissue homogenates was determined using a Total Protein Assay kit (Nanjing Jiancheng Bioengineering Institute).

### Morphological Analysis

Hematoxylin and eosin (**HE**) staining was performed on liver tissue using standard protocols previously described by Cui et al. (2020) and Amevor et al. (2021). The tissues (µm/g) were fixed for 24 h and embedded in paraffin. Thereafter, tissue sections were selected for histological and morphological observations. Frozen sections of the liver tissue were carbowax-embedded, oil-red staining was performed, and then all the sections were viewed under a fluorescence microscope and photos were taken (DP80 Digital, Olympus, Tokyo, Japan).

### RNA Isolation and Quantitative Real-Time PCR

Total RNA was isolated from the liver, spleen, and testicular tissues using TRIzol reagent (Takara, Japan) following the manufacturer's instructions, and the concentration and purity were determined using a Nanodrop 2000C (Thermo Fisher Scientific, Waltham, MA) using the A260/280 absorbance ratio. First-strand cDNA was synthesized using a PrimeScript RT Reagent Kit (Takara, Dalian, China) according to the manufacturer's protocol. Quantitative real-time PCR (**qRT-PCR**) was performed using a CFX96 Real-time System (Bio-Rad, Hercules, CA). Each qRT-PCR reaction was performed with the volumes of 15 μL containing 6.25 μL TB GreenTM Premix (Takara), 0.3 μL forward and reverse primers, 1.5 μL cDNA and 6.65 μL DNase/RNase-Free Deionized Water (Tiangen, Beijing, China). *GAPDH* was used as an endogenous control to normalize gene expression. The fold change in gene expression was quantified using the 2^^−ΔΔCt^ method (Livak and Schmittgen, 2001) where ΔCt = Ct target gene − Ct housekeeping gene, and ΔΔCt = ΔCt - ΔCt reference. Gene specific primers used for qRT-PCR analysis were designed using Primer 5 software according to the coding sequence of the target genes. The primers used for qRT-PCR analysis are presented in Table 2.

**Table 2:** Primers used for quantitative real-time PCR (qRT-PCR).

Gene	Sequence (5’-3’)	Product length (bp)	Annealing temperature (°C)	Accession number
*Bcl- 2*	F: ATCGTCGCCTTCTTCGAGTT R: ATCCCATCCTCCGTTGTCCT	150	59	Z11961.1
*Bax*	F: GTGATGGCATGGGACATAGCTC R: TGGCGTAGACCTTGCGGATAA	90	58	XM_422067.4
*IL-10*	F: GGAGAGAGCGGAGGTTTCG R: TCCCGTTCTCATCCATCTGC	118	59.86	XM_025143715.1
*INF-γ*	F: GCTCCCGATGAACGACTTGA R: TGTAAGATGCTGAAGAGTTCATTCG	150	59	NM_205149
*IL-2*	F: GCTTATGGAGCATCTCTATCATCA R: ACTCCTGGGTCTCAGTTGGT	130	58	NM_204153.1
*IL-1β*	F: GGAGAGAGCGGAGGTTTCG R: TCCCGTTCTCATCCATCTGC	118	59.86	XM_025143715.1
*AR*	F: CTACGTCGTCAAGTGGGCAA R: TTTGTGCATCCGGTACTCGT	193	60.32	NM_001040090.
*Pgk2*	F: GTGCTAATCCTGCAAACGGC R: CGATGGGCAGTTCCAAAAGC	190	60.18	NM_204985.3
*Cyclin A1*	F: GAA ATA CAG GCC CAA GCC CT R: TCA CCG CCA AGT ACA GTG TC	137	60	XM 015278427.2
*Cyclin A2*	F: TAT GCT GCT CGC ATC GAA GT R: GGA ACT GGT TGA TCG TCG GA	164	58	NM 205244.2
*GAPDH*	F: TCCTCCACCTTTGATGCG R: GTGCCTGGCTCACTCCTT	144	60	NM_204305.1

### Statistical Analysis

All experimental data are presented as mean *±* standard deviation (**SD**). Analyses were performed with one-way analysis of variance (**ANOVA**) followed by Tukey's test using GraphPad Prism 5 (GraphPad Software Inc., San Diego, CA). Values were considered significantly different at *P* < 0.05. To fit a normal distribution, the percentages were transformed into arc sines of the square roots. Other data were transformed into common logarithms. Transformations were performed before ANOVA. Thus, the experimental data were first tested for normal distribution, and on this basis, we carried out ANOVA, in which the statistics included the homogeneity test of variance.

## RESULTS

### Effects of Dietary Combination of Quercetin and Vitamin E on Production Performance in Aged Male Chickens

The results showed that there was no difference in feed intake and weight gain among the groups (Table 3, *P* > 0.05). In addition, we observed that the spleen weight and immune organ index were significantly higher in all the treatment groups (Quercetin, Vitamin E, and Q+VE groups) than in the control group (Table 3, *P* < 0.05), however, the combination group (Q + VE) had a higher spleen weight and immune index than the Quercetin group (*P* < 0.05). No difference was observed between the Vitamin E and the combination (Q + VE) groups (*P* > 0.05, Table 3).

**Table 3:** Effects of dietary combination of Quercetin and Vitamin E on production performance of aged male chickens.

Parameters	Control	Quercetin	Vitamin E	Q + VE
Feed intake (g)	108.40 ± 1.25^a^	108.37 ± 1.26^a^	108.42 ± 1.33^a^	108.25 ± 1.30^a^
Weight gain (g)	3,438.0±323.5^a^	3,410.6±319.4^a^	3,513.94±319.8^a^	3,428.0 ±324.8^a^
Spleen wet weight (g)	3.99 ± 0.62^c^	5.63 ± 0.38^bc^	7.39 ± 1.72^ab^	9.79 ± 0.61^a^
Immune organ index^1^	1.17 ± 0.30^c^	1.63 ± 0.18^bc^	2.08 ± 0.37^ab^	2.62 ± 0.19^a^

a,b,c Means in the same row without a common superscript letter differed statistically (*P* < 0.05).

1 Immune organ index (Splenic index) = Organ weight/body weight.

### Effects of Dietary Combination of Quercetin and Vitamin E on Semen Quality of Aged Male Chickens

Sperm quality analysis showed that semen volume was similar among all groups (*P* > 0.05, Table 4). However, we observed that the treatment group showed a higher sperm count, sperm motility, and percentage motility than the control group (*P* < 0.05), however, no difference was observed in sperm count, motility, and percentage motility among the treatment groups (*P* > 0.05, Table 4).

**Table 4:** Effects of dietary combination of Quercetin and Vitamin E on semen characteristics of aged male chickens.

Parameters	Control	Quercetin	Vitamin E	Q + VE
Sperm volume (mL)	0.41 ± 0.6^a^	0.43 ± 0.29^a^	0.50 ± 0.50^a^	0.43 ± 0.10^a^
Sperm count	22.20±6.52^b^	31.67±5.63^a^	31.47 ±4.88^a^	34.13 ±6.93^a^
Sperm motility	6.83 ± 029^b^	9.00 ± 0.50^a^	9.17 ± 0.29^a^	9.00 ± 0.50^a^
% Motility rate	68.33 ± 2.89^b^	90.00 ± 5.00^a^	91.67 ± 2.89^a^	90.00 ± 5.00^a^

a,b Means in the same row without a common superscript letter differed statistically (*P* < 0.05).

### Effects of Dietary Q+VE on Serum AST and ALT Levels and TG and TC Levels in Serum and Liver of Aged Male Chickens

**Figure 2:**
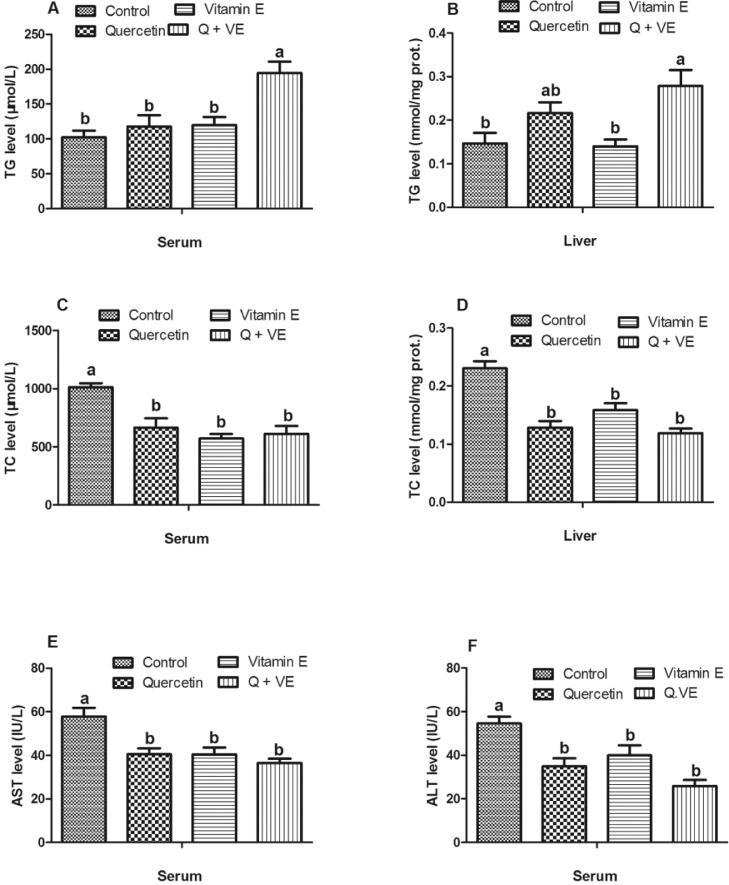
Effects of dietary quercetin and Vitamin E on serum and liver biochemical indices in aged male breeders. (A) Serum TG, (B) Liver TG, (C) Serum TC, (D) Liver TC, (E) Serum AST,(F) Serum ALT. The bar values are presented as the mean ± SD. Bars without the same letter differed significantly (*P* < 0.05).

The serum triglyceride (**TG**) level in the combination group (Q + VE) was significantly higher than that in the Control, Quercetin, and Vitamin E groups (*P* < 0.05, Figure 2A); however, no difference was observed among the Control, Quercetin, and Vitamin E groups (*P* > 0.05). In addition, for liver TG levels, the Q + VE group had a higher level of TG than the Control and Vitamin E groups (*P* < 0.05), but there was no significant difference between the Control and Vitamin E groups as well as between the Quercetin and combination groups (*P* > 0.05, Figure 2A). The levels of total cholesterol (**TC**) in the blood and liver sera decreased significantly in the Quercetin, Vitamin E, and Q + VE groups (*P* < 0.05, Figure 2B). Moreover, the levels of serum AST and ALT in the treatment groups were significantly lower than those in the control group (*P* < 0.05, Figures 2C and 2D).

### Effects of Dietary Combination of Quercetin and Vitamin E on Reproductive Hormones and Serum Immunoglobulins in the Aged Male Chickens

**Figure 3:**
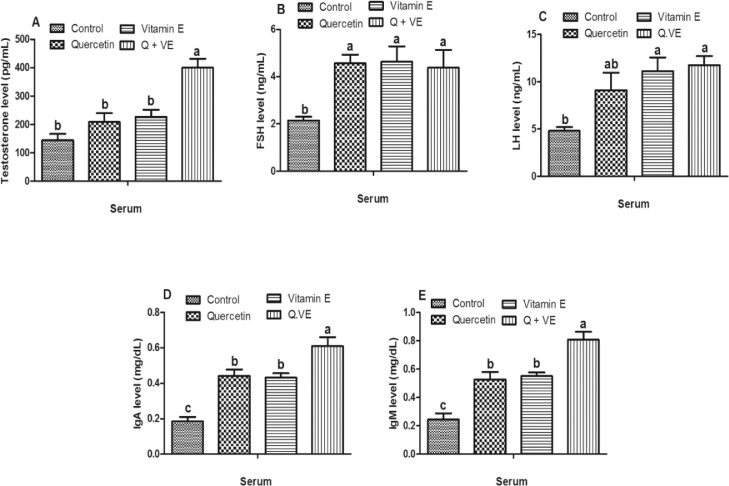
Effects of Quercetin, Vitamin E, and their combination (Q + VE) on reproductive hormones and serum immunoglobulins in aged male breeder chickens. The bar values are presented as the mean ± SD. Bars without the same letter differed significantly (*P* < 0.05).

The results showed that testosterone levels in the combination (Q + VE) group were higher than those in the Control, Quercetin, and Vitamin E groups (*P* < 0.05, Figure 3A). Furthermore, the levels of follicle-stimulating hormone (FSH) and Luteinizing hormone (LH) in the treatment groups were higher in the Q+VE as compared with the control group (*P* < 0.05, Figures 3B and 3C) except for the Quercetin group that showed no difference in LH compared with the control group (*P* > 0.05). Furthermore, serum immunoglobulin results showed that the treatment groups had increased IgA and IgM levels compared to the control group (*P* < 0.05, Figures 3D and 3E). Moreover, the combination (Q + VE) group had higher IgA and IgM levels than the Quercetin and Vitamin E groups (*P* < 0.05). There was no significant difference between the Quercetin and vitamin E groups (*P* > 0.05).

### Effect of Dietary Combination of Quercetin and Vitamin E on Antioxidant Capacity and MDA Levels in Aged Male Chickens

**Figure 4:**
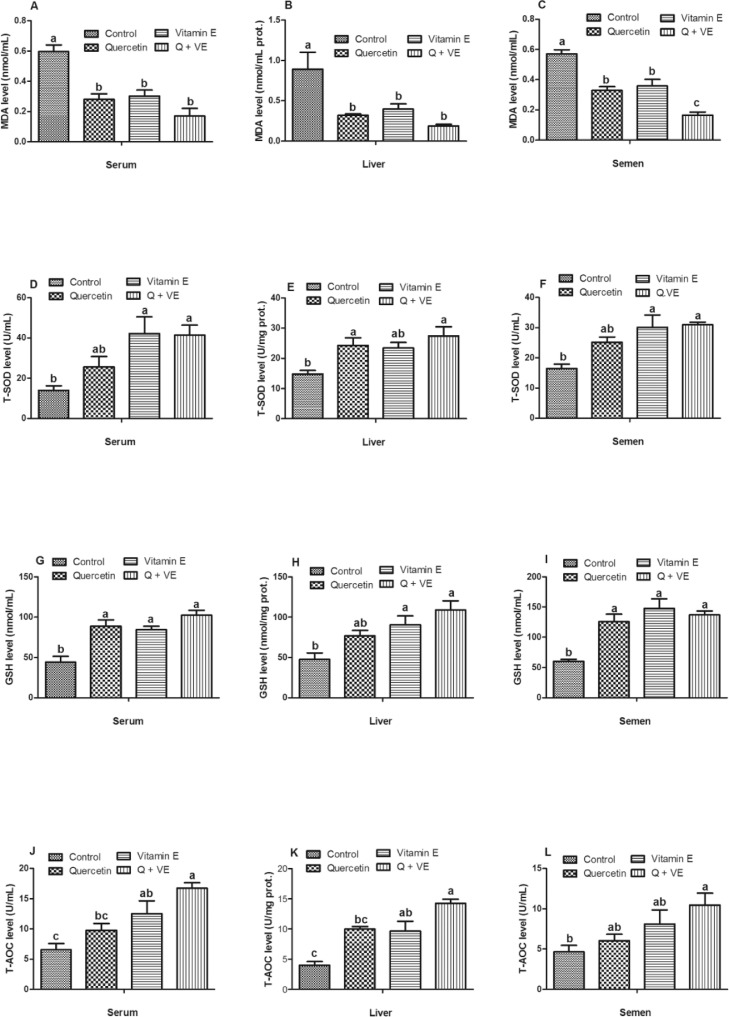
Effects of Quercetin, VE, and their combination (Q + VE) on antioxidant capacity and MDA levels in aged male breeder chickens. The bar values are presented as the mean ± SD. Bars without the same letter differed significantly (*P* < 0.05). prot: protein.

The results for the antioxidant parameters showed elevated levels of MDA in the serum, liver, and semen of the control group compared to the treatment groups (*P* < 0.05, Figures 4A–4C). T-SOD levels in the serum and semen were elevated in the treatment groups (except for Quercetin) compared with those in the control group (*P* < 0.05, Figures 4D and 4F). Similar trends were observed in the liver (*P* < 0.05, Figure 4E). However, the vitamin E group was similar to the control group (*P* > 0.05). Similarly, GSH levels in the serum and semen were significantly elevated among all dietary supplementation groups compared to the control group (*P* < 0.05, Figures 4G and 4I). GSH activity in the liver increased in all treatment groups (except for Quercetin group) compared to that in the control group (*P* < 0.05, Figure 4H). Additionally, the levels of total antioxidant capacity (**T-AOC**) in the serum, liver, and semen of the combination (Q + VE) group were significantly higher than those of the control group (*P* < 0.05, Figures 4J–4L).

### Effects of Dietary Combination of Quercetin and Vitamin E on the Expression of Spermatogenesis Related Genes in the Testes of Aged Male Chickens

**Figure 5:**
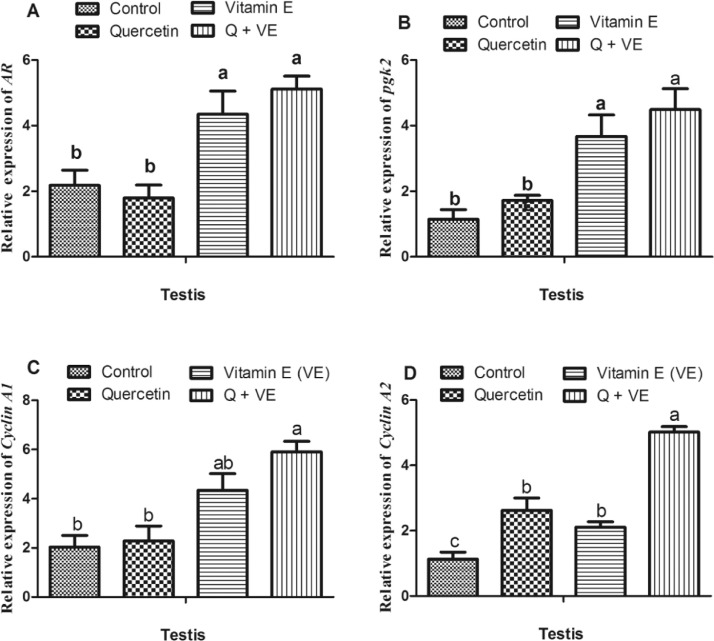
Effects of Quercetin, VE, and their combination on mRNA expression of spermatogenesis related genes in the testes of aged male chickens. The bar values are presented as mean ± SD. Bars without the same letter differed significantly (*P* < 0.05).

The mRNA expression of spermatogenesis related genes showed that the androgen receptor (***AR****)* and phosphoglycerate kinase (***pgk2****)* were significantly higher in the Vitamin E and combination (Q + VE) groups than in the control and quercetin groups, wherea*s Cyclin A1* and *Cyclin A2* were highly expressed in the combination group compared to the Control, Quercetin, and Vitamin E groups (*P* < 0.05, Figures 5A–5C), except that the expression of *Cyclin A1* was similar in the combination and vitamin E groups (*P* > 0.05).

### Effects of Dietary Combination of Quercetin and Vitamin E on the Expression of Apoptotic Related Genes in the Testes and Liver of Aged Male Chickens

**Figure 6:**
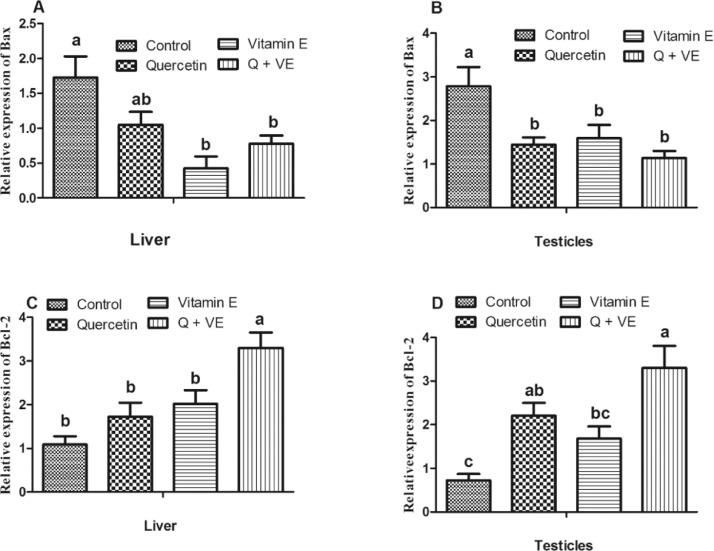
Effects of Quercetin, VE, and their combination (Q + VE) on mRNA expression of apoptotic related genes in the testes and liver of aged male chickens. The bar values are presented as the mean ± SD. Bars without the same letter differed significantly (*P* < 0.05).

The mRNA expression of apoptosis-related genes showed that the levels of *Bax* decreased in the treatment groups (except for that in the liver of the Quercetin group) compared to those in the control group in both the liver and testes (*P* < 0.05, Figures 6A and 6B). No differences were observed among the treatment groups. *Bcl_2_* levels in the liver were higher in the combination group than in the Control, Quercetin, and Vitamin E groups (*P* < 0.05, Figure 6C). However, *Bcl_2_* expression levels in the testes of the treatment groups were significantly higher than those in the testes of the control group (*P* < 0.05, Figure 6D). However, no differences were observed between the control and vitamin E groups; quercetin and combination (Q + VE) groups; and quercetin and vitamin E groups (*P* > 0.05).

### Effects of Dietary Combination of Quercetin and Vitamin E on mRNA Expression of Immune Related Genes in the Spleen and Liver of Aged Male Chickens

**Figure 7:**
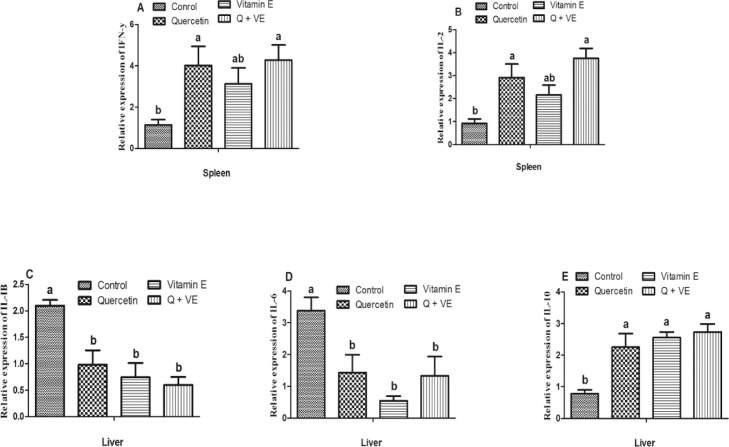
Effects of Quercetin, VE, and their combination on mRNA expression of immune related genes in the spleen and inflammation related genes in the liver of aged male chickens. Bars without the same letter differed significantly (*P* < 0.05).

The gene expression levels of *IFN-γ* and *IL-2* were higher in the treatment groups than in the control group (*P* < 0.05, Figures 7A and 7B), but there was no difference between the control and Vitamin E groups or among the treatment groups (*P* > 0.05). Moreover, for the expression of inflammation related genes in the liver, we observed that the levels of pro-inflammatory cytokines (*IL-1β* and *IL-6*) were lower in the treatment groups than in the control group (*P* < 0.05, Figures 7C and 7D). In contrast, the anti-inflammatory gene (*IL-10*) was elevated in the treatment groups compared to that in the control group (*P* < 0.05, Figure 7E). No significant differences were observed among the treatment groups for any of the inflammation related genes evaluated (*P* > 0.05).

### Effects of Dietary Combination of Quercetin and Vitamin E on Liver Morphology of Aged Male Chickens

**Figure 8:**
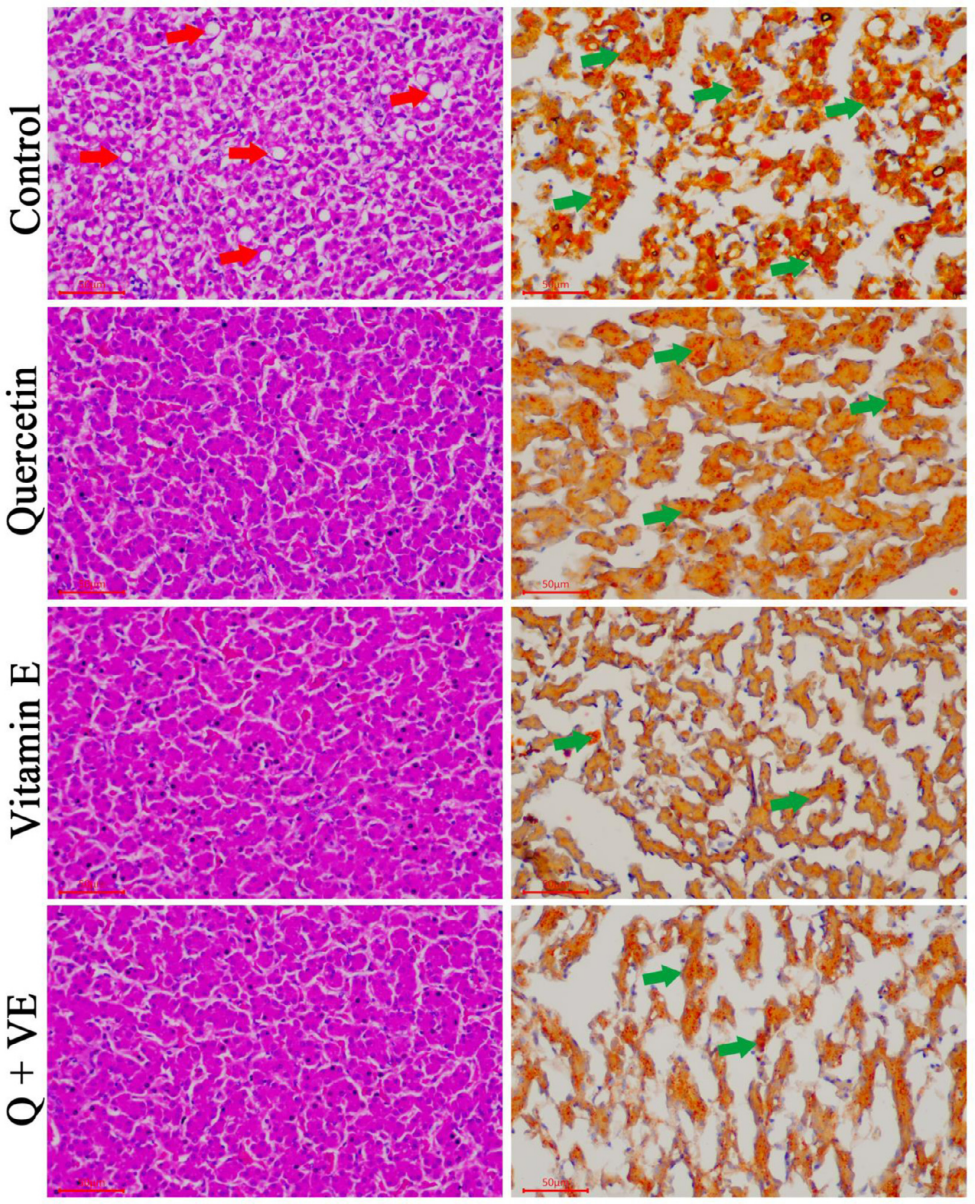
Effects of Quercetin, Vitamin E, and their combination (Q + VE) on liver morphological and histological changes of aged male chickens. This figure shows the Hematoxylin-eosin (HE) staining and oil-red results of the livers. Control Group: severe hepatic steatosis. Quercetin Group: mild hepatic steatosis. Vitamin E Group: moderate hepatic steatosis. Q + VE Group: normal liver morphology without any steatosis. The red arrow indicates the “fatty vacuoles”, the green arrow indicates the “lipid droplets”.

The histopathology and oil red staining results are shown in Figure 8. Severe pathological changes were observed in the liver of the Control group (Figure 8A), whereas, mild (Figure 8B), moderate (Figure 8C), and no (Figure 8D) pathological changes were observed in the quercetin, vitamin E, and Q+VE groups.

**Figure 9:**
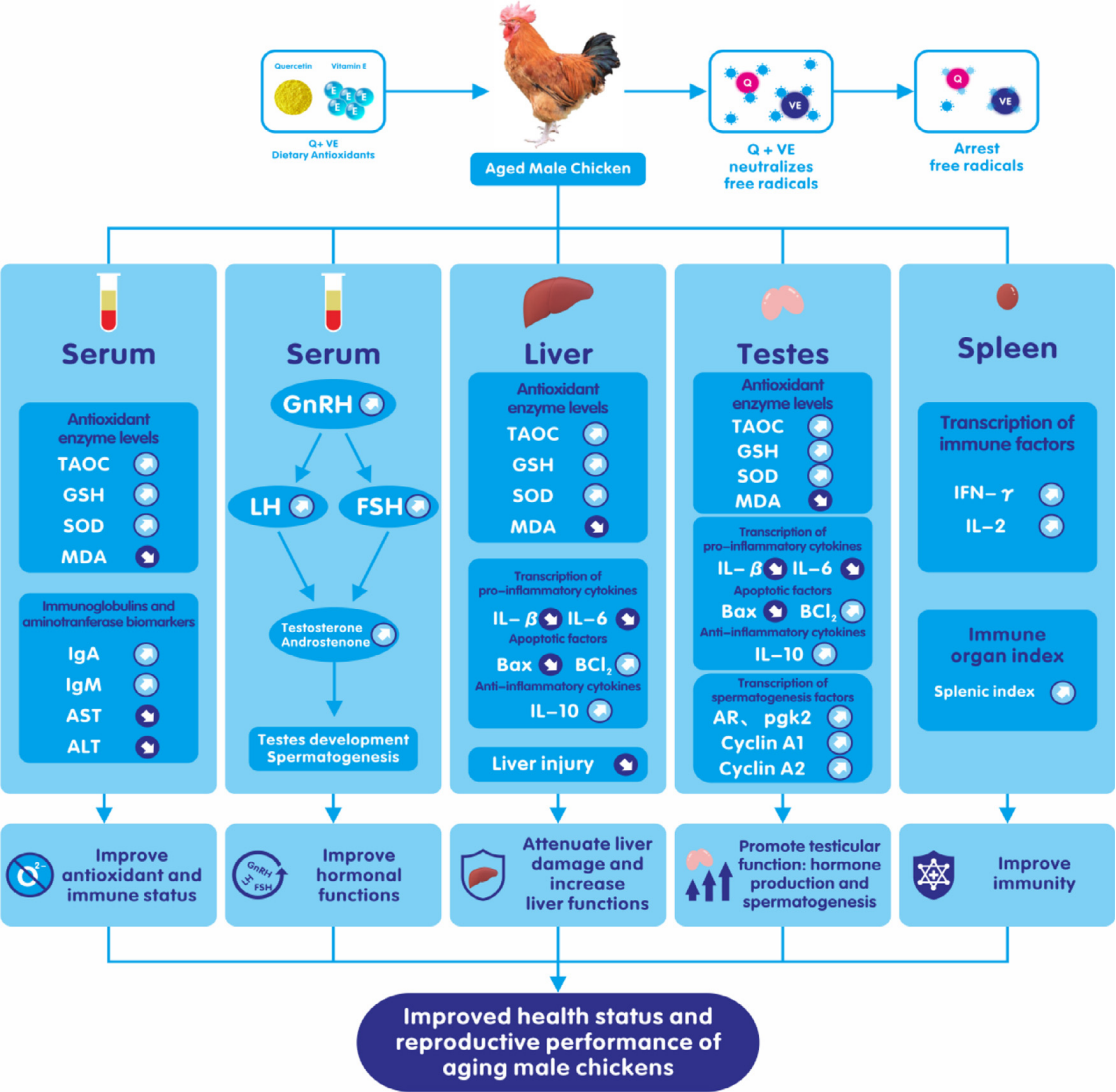
General mechanisms of action of the combination of quercetin and vitamin E on the reproductive performance of aged male chickens. The action of the combination of Quercetin and Vitamin E decreases the risk of oxidative stress to promotes fertility in aged male chickens. Combination of Quercetin and Vitamin E most likely acts via the oxidative mechanism of action to prevent oxidative stress, promote reproductive hormone production, immunity, and liver functions. These together, improve spermatogenesis, and hence, fertility and reproductive performance and health in aging male chickens.

All results obtained in this study have been summarized in Figure 9.

## DISCUSSION

In the present study, feed intake and body weight of aged male breeder chickens were not affected by supplementation with a combination of quercetin and vitamin E. Our results were consistent with those of Yang et al. (2020) who reported that dietary quercetin had no significant effect on the growth performance of chickens (Yang et al., 2020). However, these findings contradict those of previous studies that showed that supplementation with either quercetin or vitamin E produced positive effects on the body weight of broiler chickens (Maini et al., 2007; Zhang and Kim, 2020). The difference between our study and previous studies may be related to the age differences in the chickens used.

The immune organ index indicates lymphoid cell activation to produce immune factors in response to stimulation (Jeurissen, 1991; Shu et al., 2020). Our results showed that the combination of quercetin and vitamin E supplementation exerted synergistic effects on splenic weight and splenic organ index by stimulating the development of the spleen, hence improving the immune performance in aged male chickens. This finding coincided with the findings of Yang et al. (2020) who reported that quercetin could significantly increase the weights of the spleen and thymus and their indices.

Sperm abnormalities usually indicate alterations in spermatogenesis that can be attributed to age, nutrition, and pollution (Tarif et al., 2013). Our results showed that the dietary combination of quercetin and vitamin E improved sperm quality by increasing sperm count and motility. This is consistent with the findings of Khaki et al. (2010); Khan et al. (2012); Nawab et al., 2019; Najafi et al. (2020), and Khaki et al., 2010 who demonstrated that supplementation with either vitamin E or quercetin could improve the sperm quality in poultry. Vitamin E provides biological stability to the spermatozoal plasma membranes in male birds by preventing the generation of reactive oxygen species (Nawab et al., 2019). Furthermore, studies have reported that the decline in sperm quality in males may be associated with an increased level of obesity-related lipids such as cholesterol in the body (Schisterman et al., 2014). Our results showed that the combination of quercetin and vitamin E reduced the total cholesterol levels and increased the triglyceride levels in aged male chickens. This result was consistent with the findings of Pei et al. (2021), Jeong et al. (2012, 2005), and Hidiroglou et al. (2004) who reported that Quercetin and vitamin E played a vital role in reducing cholesterol levels in animals. Additionally, the liver and reproductive systems interact in a multifaceted bidirectional manner. The liver senses the body's metabolic status and adjusts energy homeostasis in a sex-dependent manner (Grossmannn et al., 2019). Many studies have reported that a reduction in reproductive function is associated with liver injury or dysfunction (Polyzos et al., 2013; Bubnov et al., 2017; Mueller et al., 2020). Therefore, the liver also improves male reproductive performance. This study revealed that the dietary combination of quercetin and vitamin E reduced the levels of AST and ALT in the serum, indicating normal liver function. This result is consistent with those of Zhu et al. (2017) and Mahmood et al. (2003) who reported a reduction in AST and ALT levels after individual quercetin and vitamin E administration in poultry. The TC, TG, AST, and ALT levels obtained in this study did not fall within the normal reference range reported by Aengwanich et al. (2004) and Cynthia et al. (2010), this may be because the normal levels of avian serum hematologic and biochemical (indices) reference vary based on several factors such as age, sex, diet, and other factors (Albokhadaim, 2012).

Hormonal regulation is an important factor in the promotion of reproduction in male animals. Gonadotropin-releasing hormone (**GnRH**) induces the secretion of luteinizing hormone (**LH**) and follicle stimulating hormone (**FSH**) from the anterior pituitary gland (Ascoli and Puett, 2009). In males, gonadotropins (FSH and LH) induce testosterone production in coordination. Testosterone further stimulates the development of testes and spermatogenesis, and maintains secondary sexual characteristics in animals (Jaros et al., 2005). Testosterone is an androgenic steroid secreted by the testes and plays an important role in the regulation of male reproduction (Zirkin and Chen, 2000). In addition, LH and FSH have been reported to be associated with testosterone and androsterone production, respectively (Quaresma et al., 2017). The results of this study showed that the combination of Quercetin and Vitamin E enhances the production of reproductive hormones (Testosterone, LH, and FSH) Selvakumar et al. (2018). reported that quercetin could improve the production of progesterone in Polychlorinated biphenyl (**PCBs**)-exposed rats.

High levels of polyunsaturated fatty acids (**PUFAs**) in avian spermatozoa render these cells vulnerable to the deleterious effects of lipid peroxidation, which are correlated with male fertility (Khan et al. 2012). Poor sperm quality has been linked to the excessive production of ROS and oxidative stress as a result of lipid peroxidation in semen (Gomez et al., 1998; Agarwal et al., 2003). Consistent ROS production is detrimental to the fertilization ability of sperm cells (Sikka, 2001, Surai, Kochish, Romanov and Griffin, 2019; Agarwal et al., 2003). The results of this study showed that the combination of Quercetin and Vitamin E reduced the levels of MDA and increased the levels of SOD, GSH, and T-AOC in serum, liver, and semen. Various studies have reported the antioxidant activities of Quercetin and Vitamin E in chickens (Breque et al., 2006; Mazur-Kuśnirek et al., 2019; Surai et al., 2019; Dong et al., 2020; Zhang and Kim, 2020).

In this study, the expression of genes related to testosterone biosynthesis and metabolism, such as *AR, pgk2, Cyclin A1, and Cyclin A2*, was high in aged male chickens that were fed a combination of Quercetin and Vitamin E. Since these genes were associated with testosterone biosynthesis (Payne and Hales, 2004; Ye et al., 2011), therefore, the upregulation of these genes and the significant increased in serum testosterone, LH, and FSH concentrations observed among the aged male chickens fed the dietary combination of Quercetin and Vitamin E showed that the supplementation of quercetin and vitamin E could synergistically promote spermatogenesis. The interplay between Sertoli and Leydig cells regulates sperm cell division and maturation under the influence of Testosterone, FSH and LH. Reports show that *Scp3* (Arango et al., 2013) and *Cyclin A1*, and *Cyclin A2* (Ravnik and Wolgemuth, 1999) are involved in the regulation of spermatogenesis. In this study, after quercetin + vitamin E feeding, spermatogenesis was significantly improved in aged male chickens, and the expression of *AR, pgk2 Cyclin A1, and Cyclin A2* was upregulated. This suggests that Quercetin + Vitamin E acts synergistically to promote spermatogenesis in aged male breeder chickens.

ROS play an important role in modulating signaling pathways for various cellular events, such as cell proliferation and apoptosis (Laurent et al., 2005). Our results showed that the dietary combination of Quercetin and Vitamin E reduced the mRNA levels of apoptosis related genes such as *Bax* and increased the expression of antiapoptotic genes including *Bcl_2_* in both the liver and testes. These results are consistent with the results obtained for the antioxidant parameters of the dietary combination of Quercetin and Vitamin E. Therefore, these results revealed that the dietary combination of Quercetin and Vitamin E would prevent apoptosis by reducing oxidative stress.

The immune system is one of the major factors that influence the health and performance of animals. In the poultry industry, chickens are continuously exposed to a wide range of stressors, that decrease growth performance, and suppress immune responses (Work et al., 2015; Yasuma et al., 2016; Yang et al., 2020). Immunoglobulins such as IgM, IgA, and IgG act as antibodies by regulating immune homeostasis. IgM is the first point of defense against harmful substances and is more abundant than IgG. In external secretions, IgA is regarded as the main immunoglobulin, hence, it is referred to as secretory IgA (Work et al., 2015; Yang et al., 2020). In this study, the dietary combination of Quercetin and Vitamin E increased serum IgA and IgM levels in aged male chickens, which is consistent with the findings on the immune organ index. This is in agreement with Yang et al. 2020, who reported that Quercetin can improve the immune function of chickens (Yang et al., 2020). Similarly, Liu et al. (2019) reported that Vitamin E supplementation significantly increased the concentrations of IgM, IgA, and IgG in chickens (Liu et al., 2019). Aging is closely related to inflammation (Bektas et al. 2017, 2018). Studies have reported that aging is characterized by the development of a proinflammatory state (Howcroft et al., 2013; Morrisette-Thomas et al., 2014). In this study, we observed that the dietary combination of quercetin and vitamin E reduced the mRNA levels of proinflammatory cytokines (*IL-1β* and *IL-6*) and increased the expression of anti-inflammatory cytokine (*IL-10*) in both the liver and testes. This was manifested in the morphological and histological results of the liver obtained in this study. Furthermore, the combination of Quercetin and Vitamin E increased the mRNA levels of immune related genes (*INF-y* and *IL-2*) in the spleen of aged chickens.

## CONCLUSIONS

This study revealed that the combination of Quercetin and Vitamin E has a synergistic effect on improving reproductive, antioxidant, and immune capabilities in aged male chickens. This study demonstrates that supplementation with dietary quercetin and Vitamin E could combat the decline in spermatogenesis, enhance the antioxidant potential, and increase the hepatoprotective capacity of aged male breeder chickens (Figure 9). However, further studies are required to explore the molecular mechanisms by which the combination of quercetin and vitamin E synergistically regulates semen quality, reproductive hormones, and immunity in aged male breeder chickens.
